# IL-32γ Delays Spontaneous Apoptosis of Human Neutrophils through MCL-1, Regulated Primarily by the p38 MAPK Pathway

**DOI:** 10.1371/journal.pone.0109256

**Published:** 2014-10-02

**Authors:** Isabelle Allaeys, Irina Gymninova, Charlotte Canet-Jourdan, Patrice E. Poubelle

**Affiliations:** Centre de Recherche en Rhumatologie et Immunologie (CRRI), Centre de Recherche du CHU de Québec, Département de Médecine, Université Laval, Québec, Canada; Virginia Polytechnic Institute and State University, United States of America

## Abstract

IL-32γ is a multifunctional cytokine involved in various inflammatory and auto-immune diseases in which neutrophils can affect the evolution of these diseases. To persist at inflammatory sites, neutrophils require inhibition of their rapid and constitutive apoptosis, an inhibitory effect that phlogogenic cytokines support. To date, the effects of IL-32γ on neutrophils remain unknown. We demonstrate that IL-32γ delays, in a dose-dependent manner, the spontaneous apoptosis of human blood neutrophils by activating mainly p38 MAPK through rapid p38 phosphorylation. PI3-K and ERK1/2 MAPK are also involved, but to a lesser extent. Most of cytokines that induce retardation of neutrophil apoptosis activate the expression of MCL-1 at both mRNA and protein levels. IL-32γ added to human blood neutrophils *in vitro* is associated with sustained levels of MCL-1 protein. This effect in neutrophils corresponds to a decrease of MCL-1 protein degradation without any effect on *MCL-1* mRNA levels. The sustained levels of MCL-1 induced by IL-32γ are only abrogated by the p38β MAPK inhibitor SB202190. Additionally, IL-32γ induces a reduction in caspase 3 activity in neutrophils. In conclusion, IL-32γ affects human blood neutrophils *in vitro* by increasing their survival, suggesting that this cytokine could have profound effects on the deleterious functions of neutrophils in several diseases.

## Introduction

Neutrophils are terminally differentiated cells that, in homeostatic conditions, constitutively undergo apoptosis *in vivo* and *in vitro* [1]. Circulating neutrophils can be considered as resting cells that remain in the blood a few hours only [2]. This short circulatory lifespan can greatly increase during infection and inflammation in response to secretion of cytokines and growth factors. These situations delay apoptosis of neutrophils, the lifespan of which can then be extended to a few days. Prolonged survival of neutrophils allows them to efficiently perform their functions of defence. Neutrophil apoptosis is also delayed *in vitro* by various cytokines including granulocyte-macrophage colony-stimulating factor (GM-CSF), G-CSF, interleukin-1 (IL-1), IL-4 or IL-6 [3], [4], [5], [6]. Retardation of neutrophil apoptosis by cytokines, inflammatory mediators or microorganisms could, however, lead to persistent inflammation and tissue damage induced by secretion of cytotoxic molecules such as reactive oxidants and proteases [7], [8], [9].

Since neutrophils are constitutively programmed for rapid cellular apoptosis, the earliest modification of neutrophils involved in host reactions to extracellular stimuli corresponds to the delay of their spontaneous apoptosis. However, the large *spectrum* of neutrophil functions that can lead to inflammation requires tight control of neutrophil survival and apoptosis [10], [11]. This complex control uses extrinsic and intrinsic pathways [11]. Among the extrinsic pathways are anti-apoptotic factors such as cytokines/growth factors and pro-apoptotic factors such as FasL/TRAIL. On the other hand, caspases and Bcl-2 family member proteins are the main intrinsic pathways [10]. However, most of these pathways are differentially regulated by phosphorylation/dephosphorylation states in which phosphatidylinositol 3-kinase (PI3-K) and mitogen-activated protein kinase (MAPK) cascades are mostly involved [12], [13], [14], [15].

Neutrophils express most of the pro-apoptotic caspases that are subdivided into initiator and effector caspases (respectively caspases 8, 9, 10, and 3, 6, 7) [10], [16]. In addition, caspase 3, which is highly expressed in neutrophils, represents a critical enzymatic step to induce neutrophil apoptosis by cleaving cellular proteins, nuclear DNA and NFκ-B [17], [18], [19]. Besides caspases, members of the Bcl-2 protein family also tightly regulate neutrophil apoptosis [20]. Regarding this protein family, human neutrophils express the pro-apoptotic proteins Bax, Bid, Bak, and Bad that remain stable with long half-lives, and the anti-apoptotic proteins MCL-1 (myeloid cell leukemia 1), A1 and Bcl-X that are unstable and short-lived [21]. However, human neutrophils do not express Bcl-2 or Bcl-X at the protein level [21], [22]. To date, MCL-1 is undoubtedly the most studied survival protein of neutrophils present in different *in vitro* and *in vivo* stimulatory conditions, since MCL-1 is a regulatory protein influenced by many pro- and anti-apoptotic signals [22], [23], [24]. More specifically, cytokine-activated survival of neutrophils has been shown to critically depend on cellular levels of MCL-1 [4], [22]. This major anti-apoptotic factor for neutrophils is very rapidly transcribed [25], [26]. However, cytokine-induced increases in MCL-1 seem to be regulated more at the protein level than at the mRNA level [24]. For instance, GM-CSF up-regulates MCL-1 mainly by stabilizing its expression at the protein level [27]. In addition, mature MCL-1 has a very short (<5 hr) half-life, and MCL-1 amounts have been inversely correlated to neutrophil apoptosis [28], [29]. The protein MCL-1 is characterized by several phosphorylation sites that allow tight up- and down-regulation of neutrophil survival [30]. Thus, experiments with highly purified human neutrophils suggested that at early timepoints MCL-1 decreases before caspase 3 activation, and at later timepoints reduction of MCL-1 amounts depends on caspase activity [31].

Interleukin-32 (IL-32), originally reported as natural killer (NK) transcript 4, is a newly described multifunctional cytokine mainly produced by activated cells like T lymphocytes, NK cells, monocytes and epithelial cells [32], [33], [34]. IL-32 presents pro-inflammatory properties and influences innate as well as adaptive immune responses [35], [36], [37]. There are six splice variants of IL-32 (IL-32α, IL-32β, IL-32γ, IL-32δ, IL-32ε, and IL-32ζ), among which the γ isoform has the longest sequence associated to a protein with the most efficient biological activity [38], [39]. Overexpression of IL-32γ has been associated with cell death in T lymphocytes and HeLa cells [39]. In addition, IL-32γ inhibited tumor development by interfering, at least in part, with the expression of anti-apoptotic genes [40]. This interleukin induces a variety of proinflammatory cytokines such as TNF-α, IL-1β, IL-6 or IL-8 [33], [41]. Moreover, IL-32 has been shown to be associated with various inflammatory and auto-immune pathologies such as rheumatoid arthritis, inflammatory bowel diseases and certain cancers [42], [43], [44], [45], [46], [47]. Also, neutrophils are recognized as major players in immune diseases and cancers [48], [49], [50], [51]. Thus, IL-32 appears to be a critical proinflammatory cytokine that impacts immune responses, but whether or not neutrophils can be affected by IL-32 remains unknown.

In the present investigation, we aimed to determine whether IL-32γ affects survival of human neutrophils. We demonstrated that IL-32γ significantly delays the spontaneous apoptosis of neutrophils. Studies with pharmacological inhibitors suggested the involvement of p38 MAPK and PI3-K in IL-32γ anti-apoptotic signals resulting in MCL-1 stabilization and caspase-3 inhibition. These data indicate that IL-32γ, by increasing the lifespan of mature neutrophils, could be responsible for persistent inflammation and tissue damage associated with chronic inflammatory diseases.

## Materials and Methods

### Reagents and antibodies

The incubation medium RPMI 1640, FBS, penicillin/streptomycin and Ficoll were obtained from Wisent (St-Bruno, QC, Canada). Dextran was from Sigma-Aldrich Corp. (St. Louis, MO, USA). Recombinant human IL-32γ, ApoStat detection kit and TACS annexin V-FITC apoptosis detection kit were purchased from R&D Systems (Minneapolis, MN, USA). The inhibitors of PI3K/Akt (LY294002) and MEK-1 (U0126) were obtained from Cayman Chemical (Ann Arbor, Michigan, USA). The JNK inhibitor (SP600125) was from Calbiochem (San Diego, CA, USA), p38 MAPK inhibitor (SB202190) was obtained from Invivogen (San Diego, CA, USA), and MAPK inhibitor negative control (SB202474) was from Life Technologies (Carlsbad, CA, USA). The fluorescein active caspase-3 staining kit was purchased from BioVision (Milpitas, CA, USA) and the caspase-3 (active form) apoptosis kit was from BD Biosciences (San Jose, CA, USA). Antibodies were from different sources as follows: anti-human CD66b-FITC and control isotype were from BioLegend (San Diego, CA, USA), anti-human MCL-1 and anti-human PCNA (PC-10) were from Santa Cruz Biotechnologies (Dallas, TX, USA), anti-human phopho-p38 and anti-human p38 MAPK were from Cell Signaling Technologies (Danvers, MA, USA), and anti-human β-actin was from Sigma-Aldrich Corp. (St. Louis, MO, USA).

### Isolation and incubation of neutrophils

The institutional review board of the Université Laval (Québec, QC, Canada) approved this study and healthy adult volunteers signed a consent form. Peripheral blood neutrophils were prepared in sterile conditions at room temperature, as previously described [52]. Briefly, human venous blood collected on citrate phosphate dextrose adenine anticoagulant solution was centrifuged (250×*g*, 10 min) to remove the platelet-rich plasma. After dextran sedimentation of the erythrocytes, neutrophils were purified by centrifugation over a Ficoll-Paque cushion (450×*g*, 20 min). Neutrophils were collected at the bottom and contaminating erythrocytes were eliminated by hypotonic lysis. Neutrophils were counted and suspended in the appropriate medium: RPMI 1640+10% FBS for long-term incubation (≥2 hrs) or HBSS for short-term incubation (≤1 hr). In order to exclude potential monocyte contamination, cell purity was routinely assessed by fluorescence activated cell sorting (FACS) using an anti-CD66b antibody [53] and was >99.5%. Neutrophils (10^7^ cells/ml) were incubated (37°C, 5% CO_2_) with IL-32γ, pharmacological inhibitors or their vehicle for the indicated times.

### Annexin V-FITC binding evaluation

Non-apoptotic and non-necrotic neutrophils were referred to as viable cells. Neutrophil apoptosis was evaluated with the TACS annexin V-FITC apoptosis detection kit, as suggested by the manufacturer. Neutrophils were simultaneously stained with FITC-labeled annexin V and propidium iodide (PI) to discriminate between early and late apoptosis and necrosis. Briefly, 5×10^5^ neutrophils were incubated 15 min on ice in 100 µL of binding buffer containing Annexin V-FITC and PI, in the dark. Ten thousand cells were then counted and analyzed by FACS using the FACScalibur flow cytometer (BD Biosciences, San Jose, CA, USA).

### Measurement of pan-caspase activity

Cells undergoing apoptosis were irreversibly labeled with a cell permeable, FITC-conjugated pan-caspase inhibitor (ApoStat assay FITC-VD-FMK). Cells were then analyzed by flow cytometry for the presence of bound reagent. The test was performed as specified by the manufacturer. Briefly, 10^6^ neutrophils were incubated with or without IL-32γ for 48 hrs, Apostat-FITC was added directly to the cells for the last 30 min of incubation (37°C, 5% CO_2_), in the dark. Cells were then washed in PBS and analyzed by FACS. Fluorescence intensity was proportional to cells undergoing apoptosis.

### Active caspase-3 measurement

The fluorescein active caspase-3 staining kit was used to detect activated caspase-3 in living cells. The caspase-3 inhibitor, DEVD-FMK conjugated to FITC (DEVD-FMK-FITC), is used as a marker and irreversibly binds to activated caspase-3 in apoptotic cells. The test was performed as specified by the manufacturer. Briefly, 10^6^ neutrophils were incubated with or without IL-32γ for 48 hrs, DEVD-FMK-FITC was added directly to the cells for the last 30 min of culture (37°C, 5% CO_2_), in the dark. Cells were then washed twice in wash buffer and analyzed by FACS. Fluorescence intensity was proportional to cells with activated caspase-3.

The caspase-3 (active form) apoptosis kit was also used to detect apoptotic cells. The test was performed as specified by the manufacturer. Briefly, 10^6^ neutrophils were incubated with or without IL-32γ for 48 hrs. After fixation and permeabilisation steps, cells were incubated 30 min in the dark with an antibody that specifically recognizes the active form of caspase-3. Cells were then washed and analyzed by FACS. Fluorescence intensity was proportional to cells undergoing apoptosis.

### RNA isolation and RT-PCR

Total RNA of neutrophils was isolated using trizol (Life Technologies, Carlsbad, CA, USA). Briefly, 10^7^ cells stimulated or not with IL-32γ (500 ng/ml) were homogenized in 1 ml Trizol. Total RNA was then extracted, according to the manufacturer's protocol. Reverse-transcription and PCR were then performed. Firststrand cDNA synthesis was performed using 1 µg of total RNA and Superscript III (Life Technologies, Carlsbad, CA, USA) under recommended conditions, with 50 ng of random hexamers (Life Technologies, Carlsbad, CA, USA). The cDNAs for β-actin and MCL-1 were amplified by polymerase chain reaction (PCR) using gene-specific primer pairs designed with Primer 3 software (Whitehead Institute for Biomedical Research, Cambridge, MA) for β-actin: 5′-CGT GAC ATT AAG GAG AAG CTG TGC- 3′ (forward), 3′- CTC AGG AGG AGC AAT GAT CTT GAT - 5′ (reverse) and MCL-1: 5′- TGC TGG AGT AGG AGC TGG TT - 3′ (forward), 3′- CCT CTT GCC ACT TGC TTT TC - 5′ (reverse). Each PCR was performed using Taq DNA polymerase from Bio Basic (Markham, ON, Canada) and the number of cycles corresponding to the linear phase of amplification and the annealing temperature were optimized for each primer set. Amplification conditions were as follows: 95°C (20 sec), 60°C (20 sec), 72°C (30 sec); 25 cycles for actin and 26 cycles for MCL-1.

### Immunoblot analysis

Neutrophils (5×10^6^ cells) were treated with IL-32γ in the presence or absence of kinase inhibitors, and cells were harvested, washed with PBS and then directly lysed in Laemmli buffer. Samples were subjected to SDS-polyacrylamide gel electrophoresis (SDS-PAGE) and transferred to PVDF membranes (Bio-Rad Laboratories, Mississauga, ON, Canada). Equal protein loading and transfer efficiency were visualized by β-actin detection. Membranes were saturated for 40 min at room temperature in Tris-buffered saline (TBS: 25 mM Tris-HCl pH 7.6, 0.2 M NaCl) with 0.5% Tween 20, containing 5% (w/v) dried milk and subsequently incubated overnight at 4°C with anti-MCL-1, anti-phospho-p38, anti-p38 or 1 hr at room temperature with the anti-β-actin antibody, followed by incubation with secondary antibodies. Bound antibodies were revealed with Western Lightning Plus ECL, as specified by the manufacturer's protocol (PerkinElmer, Waltham, MA, USA).

### Densitometric analysis

Immunoblots and cDNA amplifications were analyzed using Image Lab software and band intensity was quantified by densitometry. Results are presented as mean values of arbitrary densitometric units normalized to the expression of β-actin.

### Statistical analysis

Results are expressed as mean ± SEM. Statistical analyses were performed using GraphPad Instat 3.0 (GraphPad Software Inc., San Diego, CA, USA). Two groups were analyzed using paired *t* tests. For three groups or more, statistical analyses were performed by using the one-way ANOVA Bonferroni multiple comparison test or the repeated measures ANOVA. Significance was set at P<0.05.

## Results and Discussion

### IL-32γ delays spontaneous apoptosis of neutrophils

**Figure 1:**
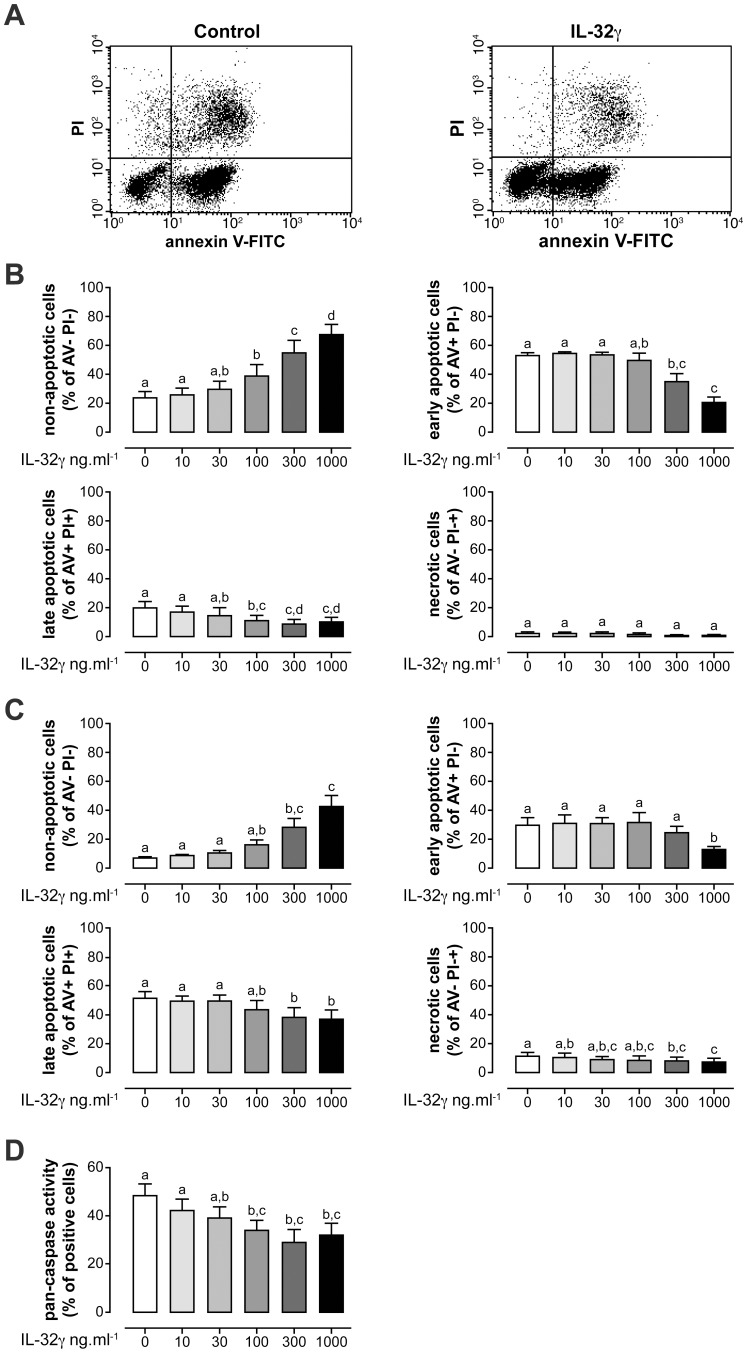
IL-32γ delays neutrophil apoptosis. Neutrophils were incubated with graded concentrations of IL-32γ or its vehicle. Cells were analyzed by flow cytometry for annexin V-FITC binding in conjunction with propidium iodide (PI) staining. Representative FACS analysis of neutrophils incubated for 24 hrs with vehicle (Control) or 300 ng.ml^−1^ of IL-32γ and stained with annexin V-FITC and PI (**A**). Neutrophils were incubated with vehicle (0) or graded concentrations of IL-32γ (10, 30, 100, 300 and 1000 ng.ml^−1^) for 24 hrs (**B**) or 48 hrs (**C**). Results are illustrated in (A) as annexin V-FITC negative and PI negative cells (non-apoptotic cells; present in the lower left quadrant of FACS), annexin V-FITC positive and PI negative cells (early apoptotic cells; lower right quadrant), annexin V-FITC positive and PI positive cells (late apoptotic cells; upper right quadrant) or annexin V-FITC negative and PI positive cells (necrotic cells; upper left quadrant). Apoptosis was also evaluated by measurement of neutrophil pan-caspase activity after 48 hrs of incubation with IL-32γ or its vehicle. Activated caspases were labelled with a FITC-conjugated pan-caspase inhibitor before being analyzed by FACS (**D**). Results represent the means ± s.e.m. of 4 different donors. Statistical significance was assessed using one-way ANOVA followed by Bonferroni multiple comparison test: means without a common letter differ (P<0.05).

Several cytokines/growth factors, such as TNF-α or GM-CSF associated with the inflammatory process delay neutrophil apoptosis [22], [54], [55]. Knowing that IL-32 contributes to inflammation in rheumatoid arthritis, inflammatory bowel disease and cancer [43], [46], [47], chronic diseases in which neutrophils are implicated, we investigated the effect of IL-32γ, the longest and the most active isoform, on neutrophil apoptosis. Evaluation of apoptosis was performed using two techniques that assess different markers of apoptosis: 1/the binding of annexin V to phosphatidylserine on the outer surface of neutrophils, a well-recognized method for flow cytometric detection of early apoptotic cells [56], and 2/the binding of a labeled pan-caspase inhibitor to the active caspase enzymes. In addition to the evaluation of annexin V, membrane-permeable late-apoptotic and necrotic cells were evaluated by PI, a fluorescent dye which stains DNA by intercalating between bases and which is excluded from viable cells. Apoptosis was studied using healthy human blood neutrophils incubated in the presence or absence of graded concentrations of IL-32γ (Figure 1).

Viable neutrophils are non-apoptotic cells that are not stained by annexin V or PI. After 24 hrs of incubation with vehicle, the normal neutrophil population can be arranged into four categories characterized by different percentages that vary with experimental conditions: 23.9±3.5% non-apoptotic (annexin V and PI negative), 53.0±2.1% early apoptotic (annexin V positive and PI negative), 20.4±4.3% late apoptotic (annexin V positive and PI positive) and 2.7±1.0% necrotic (annexin V negative and PI positive) cells (Figure 1A). In contrast to vehicle, IL-32γ increased the percentage of non-apoptotic neutrophils while reducing percentages of early and late apoptotic cells without significant modification of necrotic cells, in a dose-dependent manner. Remarkably, after 24 hrs of incubation IL-32γ maintained at least twice as many neutrophils in a non-apoptotic state compared to vehicle: percentages of annexin V negative/PI negative cells decreased from 54.9±8.7% in the presence of 300 ng/ml IL-32γ and from 67.6±6.9% with 1000 ng/ml IL-32γ to 23.9±3.5% in control conditions (Figure 1B). This effect was more pronounced at 48 hrs (Figure 1C), since IL-32γ maintained neutrophils in a non-apoptotic state at least 4 times more efficiently than did vehicle: percentages of annexin V negative/PI negative cells decreased from 28.5±5.9% (incubation with 300 ng/ml IL-32γ) and from 42.5±7.9% (with 1000 ng/ml IL-32γ) to 7.2±1.2% (incubation with vehicle). A representative FACS analysis of neutrophils incubated with vehicle or IL-32γ is shown in Figure 1A. In addition, IL-32γ reduced the levels of active pan-caspase in a dose-dependent manner, which indicated an inhibitory effect of IL-32γ on neutrophil apoptosis (Figure 1D). In fact, kinetics and amplitude of the inhibitory effect of IL-32γ on neutrophil apoptosis were similar to those of other cytokines which delay neutrophil apoptosis, such as GM-CSF, IL-8 or TNF-α [22], [54], [55], [57], [58], [59]. It is also useful to stress that, in addition to pancreatic cancer cells in which IL-32 reduces cellular apoptosis [44], neutrophils are the second cell type reported to show an increase in survival in the presence of IL-32.

### IL-32γ-delayed apoptosis depends on p38 MAP Kinase and PI3-Kinase

Spontaneous (or constitutive) neutrophil apoptosis, as well as neutrophil apoptosis retarded by extracellular stimuli produced during the inflammatory process, are tightly regulated for maintaining cell homeostasis [11], [20]. Mechanisms that regulate the effects of anti-apoptotic factors involve various kinase cascades like PI3-kinases/Akt, MAP kinases, PKCδ, JNK and STAT [11], [58], [60]. Therefore, to investigate kinase pathways that could be involved in the regulation of IL-32γ-delayed apoptosis of neutrophils, pharmacological inhibitors at optimal concentrations were used to study the role of PI3-K, p38 MAPK, ERK and JNK activation in this anti-apoptotic effect.

LY294002, an inhibitor of class I PI3-K [61], inhibited the anti-apoptotic effect of IL-32γ by 51% (Table 1). U0126, a selective MEK inhibitor targeting ERK1/2 MAPK [62], [63], inhibited the anti-apoptotic effect of IL-32γ by 29%. Preincubation with SP600125, an inhibitor of JNK [64], did not affect the anti-apoptotic effect of IL-32γ. Inhibition of p38β MAPK by pretreating neutrophils with SB202190 [65] reduced IL-32γ effects on neutrophil survival by 68%. It is useful to point out that the major function of the β isoform of p38 MAPK relates to survival [66], and that SB202190 abrogated p38 MAPK without inhibiting ERK and JNK activity [67], [68]. As a control for SB202190 effectiveness, pretreating neutrophils with SB202474, the inactive analog of SB202190, did not influence IL-32γ effects on neutrophil survival (dat not shown).

**Table 1:** Effects of signaling inhibitors on IL-32γ delayed apoptosis.

	Delayed apoptosis (%)	p (n)
IL-32γ	100	
IL-32γ+LY 294002	49.1±7.2	p = 0.0001 (9)
IL-32γ+U 0126	70.8±3.2	p<0.0001 (9)
IL-32γ+SB 202190	32.1±9.9	p = 0.001 (6)
IL-32γ+SP 600125	106.7±6.1	NS (5)

IL-32γ-delayed apoptosis is dependent on kinase activation. Neutrophils were pre-incubated 30 min with an inhibitor of PI-3 kinase (LY249002 at 20 µM), an inhibitor of MEK (U0126 at 10 µM), an inhibitor of p38 MAP kinase (SB202190 at 25 µM), and an inhibitor of JN Kinase (SP600125 at 10 µM) or their vehicle (DMSO). Neutrophils were then stimulated with IL-32γ (300 ng.ml^−1^) for 20 h and further analyzed by FACS to evaluate their annexin V-FITC binding in conjunction with propidium iodide staining. The effects of signaling inhibitors were compared to the experimental condition in which neutrophils were incubated with IL-32γ + inhibitor vehicle (DMSO); this condition corresponds to 100% of delayed apoptosis. Results are expressed as percentage of IL-32γ inhibitory effect. Results are means ± s.e.m. of 5–9 different donors. Statistics: paired Student *t* test, p values are indicated in the table. NS  =  non-significant.

**Figure 2:**
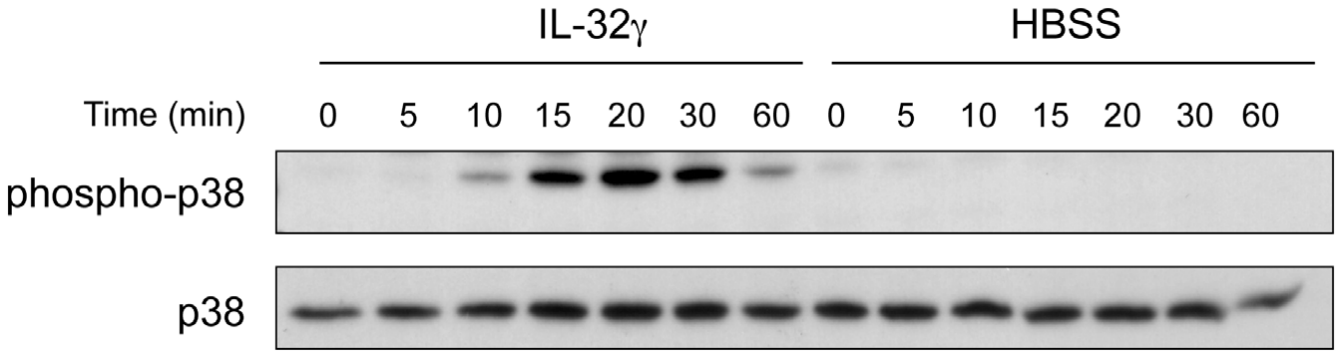
IL-32γ induces phosphorylation of p38 MAPK in human blood neutrophils. Freshly isolated neutrophils were incubated in the presence of IL-32γ (500 ng.ml^−1^) or its vehicle (HBSS) for the indicated time. Phosphorylation of p38 and total p38 were detected by immunoblot. Representative of 4 different donors.

These pharmacological results suggested that p38β MAPK could be a major kinase pathway in IL-32γ-delayed apoptosis of neutrophils. Thus, the next step was to verify whether IL-32γ could phosphorylate p38 leading to MAPK activation. As expected, IL-32γ stimulated the phosphorylation of p38 MAPK (Figure 2). Kinetics of this p38 phosphorylation took the shape of a bell curve with the onset at 10 min followed by a peak at 20–30 min and with the end at 60 min.

Interestingly, IL-32 was initially shown to increase phospho-p38 MAPK expression within 5 min followed by a decrease from 15 to 30 min and a second increase at 45 min that thereafter diminished slowly [33]. Regarding our results, the p38 MAPK pattern of human neutrophil response to IL-32γ with one peak only is similar to that seen in RAW cells, a mouse leukaemic monocyte-macrophage cell line used in the first report. Although the initial findings were obtained with IL-32α in murine immortalized myeloid cells, the phosphorylation of p38 MAPK stimulated by IL-32 appears to be a major activation pathway of this new cytokine. This rapid increase in phospho-p38 MAPK is also a hallmark of cell activation by other cytokines known to reduce apoptosis, such as GM-CSF and TNF-α [59]. In addition, the absence of involvement of the JNK MAPK pathway in IL-32γ-stimulated neutrophils could be related to the well-documented interplay between the p38 MAPK and JNK pathways in which p38 MAPK negatively regulates JNK activity [69].

### IL-32γ induces sustained amounts of neutrophil MCL-1 protein

The pro- and anti-apoptotic proteins of the Bcl-2 family are currently recognized as major regulators of neutrophil death and survival. For example, the stable pro-apoptotic protein Bax in its serine phosphorylated form heterodimerizes with the labile anti-apoptotic protein MCL-1, a condition that maintains Bax in the cytoplasm and lengthens the survival of neutrophils by reducing Bax translocation to the mitochondria [21], [70]. Moreover, MCL-1 is reported as the predominant anti-apoptotic Bcl-2 family member in neutrophils [71], [72]. Cytokines that induce a delay of neutrophil apoptosis most often depend on synthesis of new proteins by neutrophils, in particular of MCL-1 [22], [23], [29], [73]. Therefore, we investigated the effects of IL-32γ on MCL-1 expression by neutrophils at the mRNA and protein levels.

**Figure 3:**
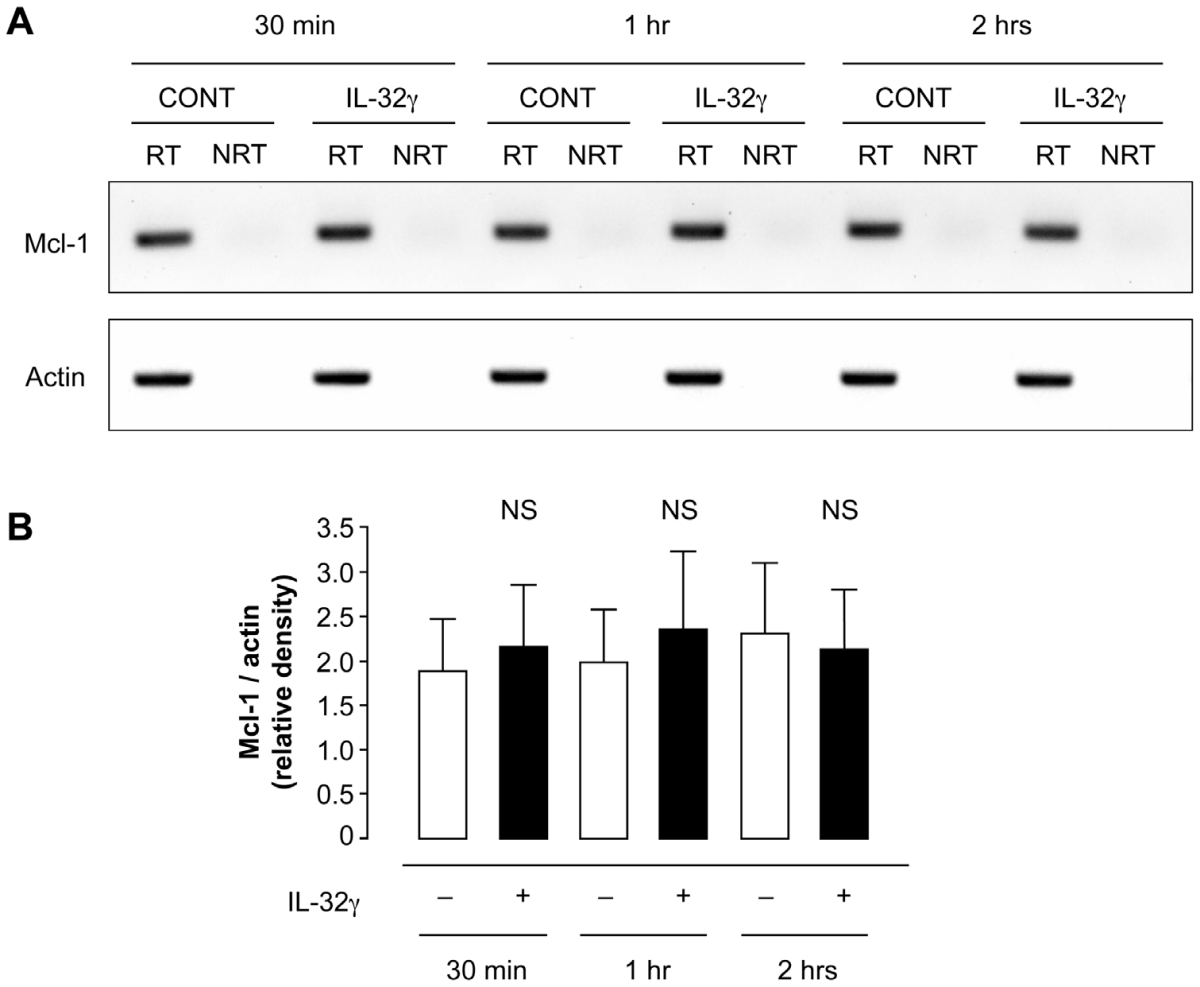
IL-32γ does not modify *MCL-1* mRNA levels. Human neutrophils were incubated 30 min, 1 hr or 2 hrs in the presence of IL-32γ (500 ng.ml^−1^) or its vehicle (CONT). After RNA extraction by using Trizol, total mRNAs were reverse transcribed (RT) or not (NRT). *MCL-1* or *actin* cDNAs were then amplified by PCR (**A**). Representative of 5 different donors. Densitometric analyses were performed and ratios between *MCL-1* and *actin* mRNAs allowed for normalization (**B**). Results are means ± s.e.m. of 5 different donors. Statistics: one-way ANOVA followed by Bonferroni multiple-comparison test (NS  =  non-significant).

**Figure 4:**
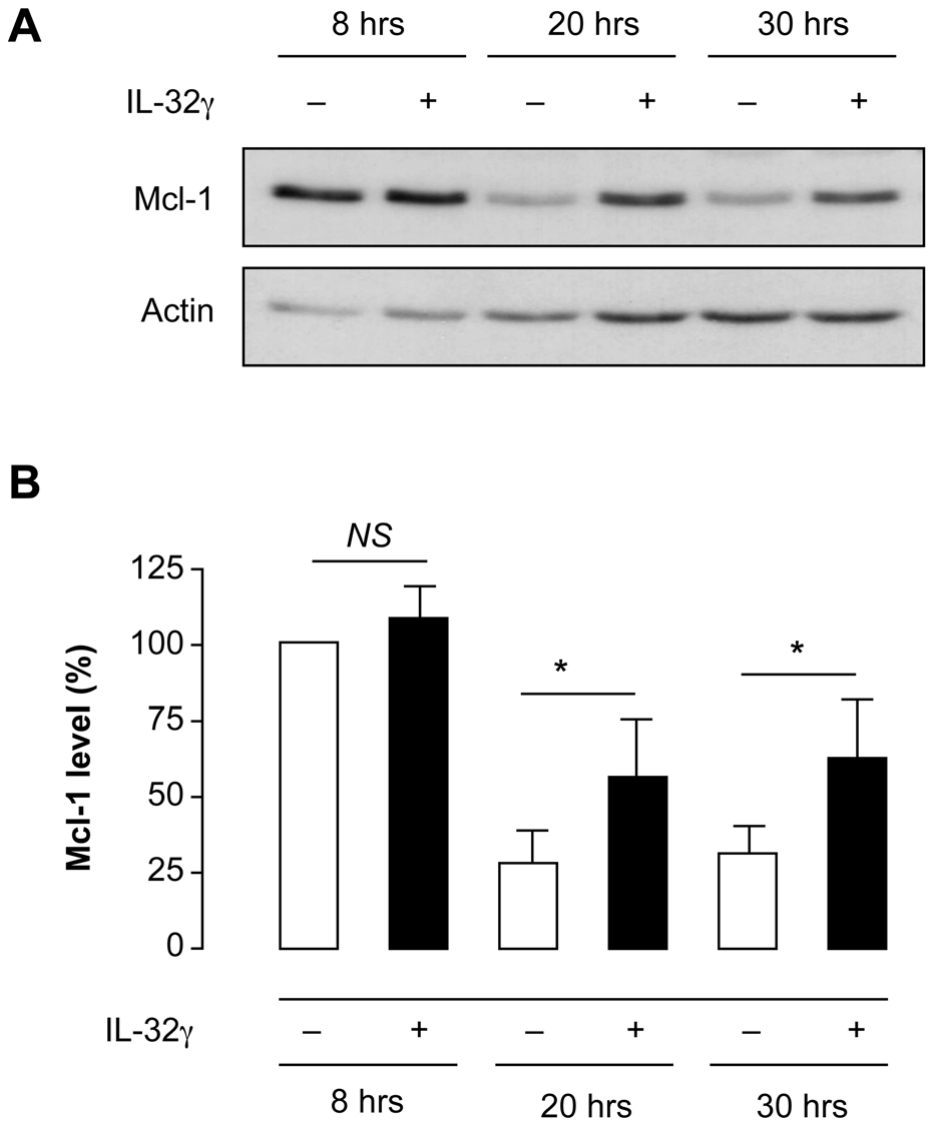
IL-32γ induces sustained steady state levels of MCL-1 protein. Human neutrophils were incubated for 8, 20 and 30 hrs with IL-32γ (300 ng.ml^−1^) or its vehicle (absence of IL-32γ). Cell lysates were subjected to SDS-PAGE and transferred to PVDF membranes. Western blotting was then performed with anti-MCL-1 or anti-β-actin (control of protein loading) antibodies, and proteins were revealed with Western Lightning Plus ECL. Representative of 4 different donors (**A**). Densitometric analyses of MCL-1 and actin expression were performed and ratios between MCL-1 and actin were calculated to normalize the data. These normalized values were then used to calculate the percentages of MCL-1 variations in neutrophils incubated with vehicle at 20 and 30 hrs of incubation or with IL-32γ at 8, 20 and 30 hrs of incubation, considering that the normalized value of MCL-1 in neutrophils incubated with vehicle at 8 hrs represents 100% MCL-1 (neutrophils at 8 hrs of incubation with vehicle were 96% non-apoptotic) (**B**). Results are means ± s.e.m. of 4 different donors. Statistics: paired Student *t* test, * p<0.05.

Neutrophils in the presence of IL-32γ did not modify their expression of *MCL-1* mRNA when compared to neutrophils incubated with vehicle for 2 hrs (Figure 3) or up to 20 hrs (data not shown). However, the addition of IL-32γ to neutrophils was associated with a sustained level of MCL-1 protein in comparison to the major decrease in MCL-1 protein levels after 20 hrs of incubation of neutrophils with vehicle (Figure 4). MCL-1 protein levels in neutrophils incubated with IL-32γ for 20 hrs were twice as high as those of neutrophils incubated with vehicle, and this effect persisted at 30 hrs of incubation (Figure 4B). On the other hand, Bcl-2 expression was under the detection limit in neutrophils incubated with vehicle or with IL-32γ (data not shown), as previously reported with other anti-apoptotic factors [21].

**Figure 5:**
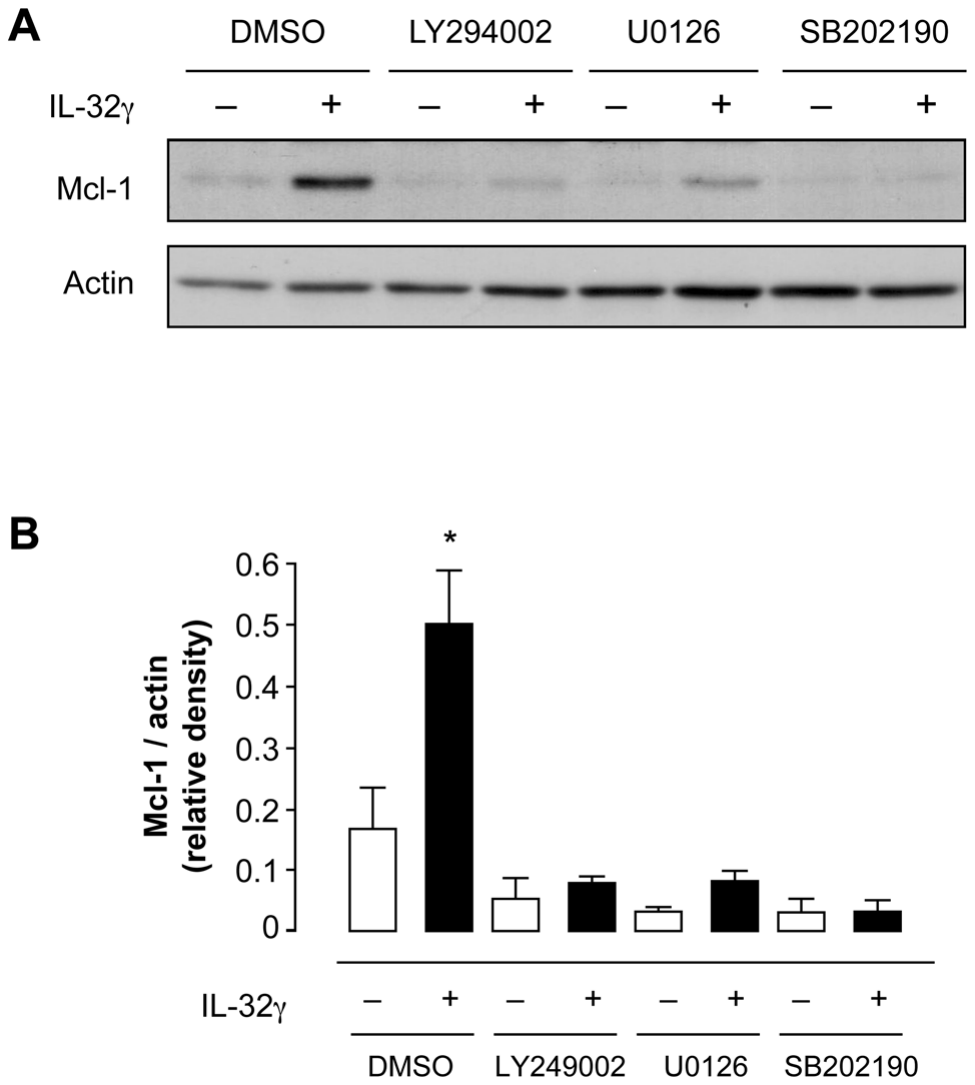
Effects of pharmacological inhibitors on IL-32γ-induced sustained amounts of MCL-1 protein. Human neutrophils were pretreated for 30 min with 20 µM LY249002 (PI-3 kinase inhibitor), or 10 µM U0126 (MEK inhibitor), or 25 µM SB202190 (p38 MAP kinase inhibitor) or their vehicle (DMSO). Neutrophils were then incubated with IL-32γ (300 ng.ml^−1^) for 20 hrs prior to cell lysis and immunoblot analysis. Immunoblot representative of 3 donors (**A**). Densitometric analyses of MCL-1 and actin expression were performed and ratios between MCL-1 and actin were calculated to normalize the data (**B**). Results are means ± s.e.m. of 3 different donors. Statistics: one-way ANOVA followed by Bonferroni multiple-comparison test, * p<0.05.

As PI3-K, ERK1/2 and p38 MAPK inhibitors modulated IL-32γ-delayed apoptosis of neutrophils (Table 1), their effects on MCL-1 expression were then investigated. All three inhibitors studied affected the basal levels of MCL-1 expression in neutrophils incubated with vehicle. However, pretreating neutrophils with the p38β MAPK inhibitor SB202190 was the sole condition that abrogated the increase of MCL-1 expression induced by IL-32γ (Figure 5). In contrast, the effects of LY294002 and U0126 were slight and non-significant. Therefore, IL-32γ delays constitutive neutrophil apoptosis by modulating MCL-1 protein amounts without affecting *MCL-1* mRNA. It is likely that IL-32γ induced an increase in steady state levels of MCL-1 by preventing its degradation. In addition, the modulation of MCL-1 amounts in neutrophils is mainly controlled by the p38 MAPK pathway, and both PI3-kinases and ERK1/2 MAPK pathways could be implicated in the regulation of anti-apoptotic proteins other than MCL-1.

To date, no report has mentioned an effect of IL-32 on MCL-1 at the protein level. The only condition in which IL-32 has been shown to affect MCL-1 was in pancreatic cancer cells that constitutively expressed IL-32α, where knockdown of IL-32α was shown to decrease *MCL-1* mRNA with no data at the protein level [44]. Interestingly, the effect of IL-32γ on neutrophil MCL-1 protein without any effect on *MCL-1* mRNA differs from that of other anti-apoptotic factors such as GM-CSF, TNF-α or LTB_4_ which were shown to upregulate the transcription of MCL-1 in neutrophils [14], [22], [24], [27], [73]. However, several reports suggest that neutrophil survival stimulated by cytokines is not mainly regulated at the transcriptional level [24]. In addition, the absence of IL-32γ effects on MCL-1 transcription in neutrophils eliminates certain activation pathways involved in survival such as NF-κB. Although IL-32 has been shown to activate the NF-κB pathway, the presence of NF-κB binding sites described in the promoter region of MCL-1 has not been demonstrated in neutrophils [30], [74], [75], [76]. Therefore, the anti-apoptotic effect of IL-32γ on neutrophils implicates a post-transcriptional modulation in which the p38 MAPK cascade appears to be the main regulator of MCL-1 protein levels. Although the role of p38 MAPK in neutrophils apoptosis seems contradictory, with pro- and anti-apoptotic effects depending on the stimulus used [12], [20], [60], [77], [78], one of the mechanisms associated with these opposing effects corresponds to the activation intensity of MAPK, and a strong p38 MAPK activity leads to apoptosis whereas a low p38 MAPK activity favors cell survival [69], [79]. Thus, our results indicate that in human neutrophils the main pathway involved in the increase in survival induced by IL-32γ depends on a rapid phosphorylation of p38 MAPK, which remains low enough to lead to post-transcriptional modulation of MCL-1. Additionally, since inhibition of PI3-K and ERK1/2 pathways did not significantly alter MCL-1 protein up-regulation but modulated the effect of IL-32γ on neutrophil apoptosis, PI3K and ERK1/2 activation could be associated with the regulation of other survival genes of Bcl-2 family members. It is useful to note that, for instance, GM-CSF can decrease the rate of MCL-1 turnover by inhibiting the proteasome, an effect regulated by PI3K and ERK signals [27]. Thus, it remains possible that IL-32γ could inhibit the proteasome leading to MCL-1 stability. However, the downregulation of neutrophil apoptosis by IL-32γ appears different from that of other cytokines such as GM-CSF or TNF-α in which PI3K and ERK pathways are strongly involved [57], [58], [80]. On the other hand, neutrophil apoptosis reduced by G-CSF has been associated with the increase in a new factor named proliferating cell nuclear antigen (PCNA) [81]. Unlike G-CSF, IL-32γ did not induce any change in PCNA as evaluated by immunoblot analysis of human neutrophils incubated *in vitro* (data not shown; n = 3).

### IL-32γ decreases caspase-3 activity in neutrophils

**Figure 6:**
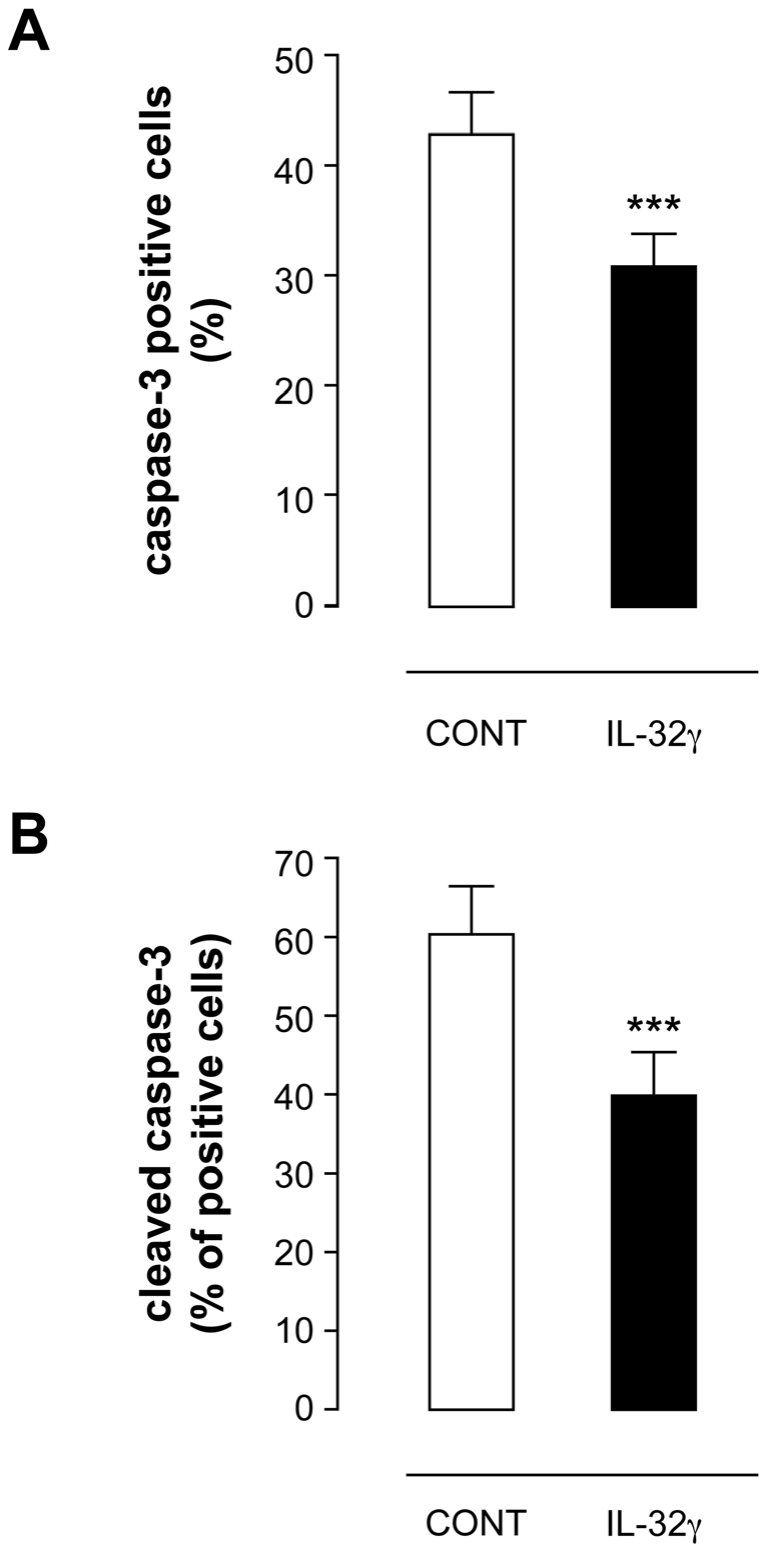
IL-32γ decreases caspase-3 activity in neutrophils. Neutrophils were incubated with IL-32γ (500 ng.ml-1) or its vehicle (CONT) for 48 hrs. Cells with activated caspase-3 were then labelled with the caspase-3 inhibitor DEVD-FMK-FITC before being analyzed by FACS. The results are means ± s.e.m. of six different donors. Statistics: paired Student *t* test (^***^ p<0.001) (**A**). Cells undergoing apoptosis were also stained with a specific antibody against the caspase-3 active form (cleaved caspase-3) before FACS analysis. The results are means ± s.e.m. of three different donors. Statistics: paired Student *t* test (^***^ p<0.001) (**B**).

Caspase 3 has been shown to be a major effector caspase in neutrophil death [17]. Interestingly, MCL-1 has recently been shown to downregulate caspase activation before being reduced by caspases themselves [31]. Thus, the increase in MCL-1 protein without mRNA change observed in neutrophils incubated with IL-32γ could be related to a reduction in caspase activity. To investigate whether IL-32γ is able to delay neutrophil apoptosis through a reduction in caspase activity, we compared the levels of active caspase-3 in neutrophils incubated with IL-32γ or with its vehicle for 48 hrs (Figure 6). The addition of IL-32γ to neutrophils reduced by 30% the number of caspase-3-positive cells from 42.9±3.7% with vehicle to 30.7±3.1% with IL-32γ, as assessed by a caspase-3 inhibitor which binds to the active enzyme (Figure 6A). Similarly, an antibody specific to the active form of caspase-3 showed a significant reduction in this pro-apoptotic enzyme in neutrophils incubated with IL-32γ (Figure 6B).

It is noteworthy that in human neutrophils p38 MAPK directly phosphorylates caspases 3 and 8 leading to decrease neutrophil apoptosis [12]. Since the major signaling pathway activated during IL-32γ-induced reduction of apoptosis and IL-32γ-stimulated increase in MCL-1 in human neutrophils is p38 MAPK (Figures 2, 5, 6), it is very likely that the anti-apoptotic effect of IL-32γ on neutrophils implicates a reduction of caspase 3 activity through its phosphorylation by IL-32γ-activated p38MAPK.

## Conclusion

To perform their functions during the inflammatory process associated with auto-immune diseases and cancers [48], [49], [50], [51], neutrophils have to persist in tissues and their survival becomes a key event of their functional condition. The pro-inflammatory cytokine IL-32γ shows a strong inhibitory effect on neutrophil apoptosis mediated through a decrease of the protein MCL-1 degradation. The present data support the fact that this sustained MCL-1 level induced by IL-32γ in neutrophils may be directly related to the effect of p38 MAPK that reduces caspase 3 activity, and thereby increases neutrophil survival. In addition, IL-32γ through PI3-K and ERK1/2 pathways could modulate anti-apoptotic proteins other than MCL-1. An important finding of the present study relates to a new mechanism through which IL-32γ could induce and perpetuate inflammation. Our data are the first demonstration that human neutrophils can respond to IL-32γ and increase their survival. Regarding the unique role of neutrophils in inflammation, the effects of IL-32γ on this cell type could be associated with tissue damage seen in various pathologies.
